# Kommerell’s diverticulum: an unusual cause of unilateral vocal cord palsy?

**DOI:** 10.1308/rcsann.2022.0092

**Published:** 2023-01-23

**Authors:** F Shaikh, D Walker

**Affiliations:** Royal Surrey NHS Foundation Trust, UK

**Keywords:** Kommerell's diverticulum, Vascular anomalies, Aberrant subclavian, Vocal cord palsy

## Abstract

Kommerell’s diverticulum is a rare congenital anomaly of the aortic arch system in which there is a left- or right-sided aortic arch with an aberrant subclavian artery on the contralateral side. Patients with this anomaly can be asymptomatic or have features of tracheal or oesophageal compression. However, there is a rising suspicion that it may be a rare cause of unilateral vocal cord palsy through its compression of the recurrent laryngeal nerve. We describe a patient who had a long history of hoarse voice and left vocal cord palsy with no other obvious cause, who was found to have a Kommerell’s diverticulum on a contrast-enhanced computed tomography scan.

## Background

**Figure 1:**
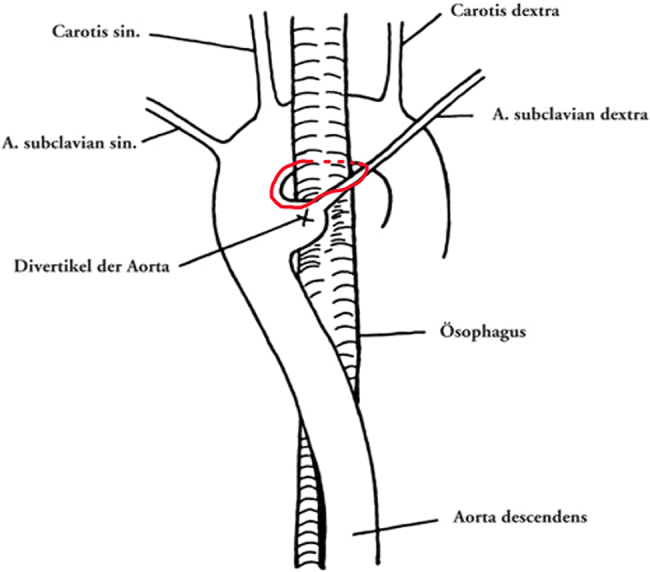
Diagram from Kommerell’s original publication illustrating the diverticulum with an aberrant subclavian artery forming a vascular ring (highlighted in red).^2^

Kommerell’s diverticulum (KD) is a rare congenital abnormality in which the fourth dorsal aortic arch fails to degenerate and gives rise to a vascular anomaly of the aortic arch system. There are multiple configurations of this anomaly; however, there are discrepancies in the literature regarding which is most common. The diverticulum refers to the outpouching of the aberrant subclavian artery at its origin from the aortic arch (Figure 1), which then forms a vascular ring around the oesophagus and trachea. Although the initial suggestion was that patients are usually asymptomatic, a review of the incidence and clinical presentation of KD by Poterucha *et al* in 2015 demonstrated that 61% of patients had symptoms.^1^ Symptoms that arise because of tracheal or oesophageal compression include dysphagia, dyspnoea, wheezing and cough. However, there has been a rising suspicion that a KD with a left-sided aberrant subclavian artery can impinge the left recurrent laryngeal nerve (RLN) and give rise to a unilateral vocal cord palsy. This case report discusses a patient that presented with left vocal cord palsy with a subsequent finding of KD, the importance of computed tomography (CT) scanning and the consideration of KD as a differential for unilateral vocal cord palsy.

## Case history

A 55-year-old woman who initially presented to the head and neck clinic 12 years ago with a long history of weak vocal quality with breathy, reduced dynamics and poor projection. This had been gradually deteriorating for the three years preceding her first presentation to clinic. Further examination, including laryngoscopy, confirmed a left vocal cord palsy with no obvious cause.

### Investigations

**Figure 2:**
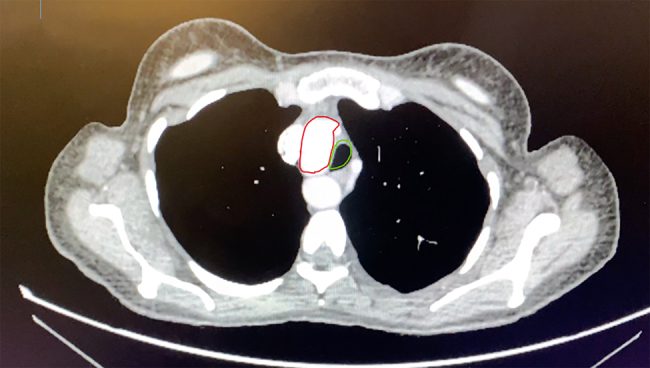
Axial contrast-enhanced computed tomography images of a 55-year-old woman (the subject of the case study) taken at approximately the level of T4. The image demonstrates a right-sided aortic highlighted in red displacing the trachea (highlighted in green) to the left.

**Figure 3:**
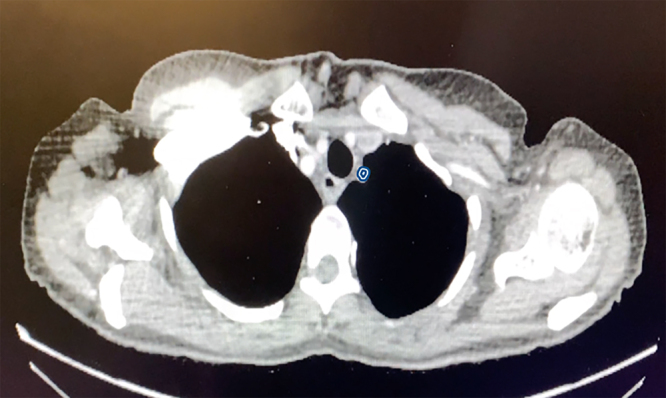
Axial contrast-enhanced computed tomography images of a 55-year-old woman (the subject of the case study) taken at approximately the level of T2. The image demonstrates a left-sided aberrant subclavian artery, highlighted in blue.

A CT scan with contrast was then performed and revealed that the patient had a right-sided four-vessel aorta with aberrant origin of the left subclavian artery (Figures 2 and 3). This formed a vascular ring around the oesophagus and trachea and the high-riding aortic arch displaced the trachea laterally to the left. In the absence of trauma, stroke, tumour or infection, it was assumed that the anatomical variant identified on imaging was a contributing factor to her left vocal cord palsy. She was subsequently referred for speech and language therapy as well as a specialist cardiothoracic opinion.

A therapeutic procedure under local anaesthetic was suggested due to the possible risk of further complications. However, with the improvement in her day-to-day life with speech and language therapy alone, she declined any surgical intervention. Following this, she was not seen in clinic for almost 10 years.

Recently with the COVID-19 pandemic and the global shift towards video conferencing, she re-presented to clinic with deteriorating symptoms. Her voice was still hoarse with a soft inspiratory stridor but there was additional concern due to a slight increase in breathlessness and noisy breathing. Flexible nasoendoscopy once again confirmed a left vocal cord palsy in a paramedian position with no obvious supraglottic impingements. A further CT scan with contrast was performed to reassess the vascular ring given the change in her symptoms in addition to extensive further investigation by a specialist cardiothoracic team at a tertiary centre.

Ultimately, she once again decided not to undergo any vascular ring surgery but is open to trying injection medialisation of her left vocal cord to improve her vocal quality. However, further assessment is required before deciding whether a local anaesthetic approach is feasible.

## Discussion

It has been 85 years since B Kommerell, a German radiologist, first made a clinical diagnosis of aberrant subclavian artery in a living patient.^2^ Kommerell’s initial description was of a left-sided aortic arch with an aberrant right subclavian artery. This variation often remains asymptomatic but those with the reverse configuration (right-sided aortic arch and aberrant left subclavian artery) may suffer from symptoms of tracheal and/or oesophageal compression. There is a discrepancy in the reported prevalence of these two configurations with some studies suggesting that Kommerell’s initial description is the more common variant.^3^ Conversely, a retrospective radiological analysis of 121 patients identified with KD demonstrated that 60% instead had a right-sided aortic arch with an aberrant left subclavian artery.^1^ The incongruity in these results is likely due to diagnostic underreporting within the literature as well as variable definitions of KD. Nonetheless, KD remains a rare diagnostic entity with a combined estimated prevalence of 0.05%–0.1%.^4^

In this case, although part of the patient’s demographic are in-keeping with reported figures,^1^ her age at presentation is unusual. Patients that present with symptoms of tracheal or oesophageal compression from a vascular ring are more commonly children, whereas adults that go on to develop symptoms later in life are often found to have an aneurysmal dilatation of KD, a Kommerell’s aneurysm. With an incidence of approximately 23%,^1^ it is a relatively common complication that is important to consider owing to the risk of rupture and subsequent morbidity and mortality. However, the change in the patient’s symptoms during her second presentation was not explained by her updated CT scan because no Kommerell’s aneurysm was demonstrated.

Her main complaint was the change in her voice due to compression of the left RLN. The RLN is a paired branch of the tenth cranial nerve (vagus) and has a long course through the neck and mediastinum after exiting from the cranial cavity. The right RLN arches around the subclavian, whereas the longer left RLN courses around the arch of the aorta and is more susceptible to compression from surrounding mediastinal structures. Left vocal cord palsies are often straightforward to assess because they require laryngoscopy or flexible nasoendoscopy. However, the aetiology can sometimes be more evasive. The most common causes are malignancy (45%) or infection (14%); however, in 8% of cases it is considered idiopathic.^5^ Before it can be labelled as such, a CT scan from the base of the skull to the costal margin of the thorax should be completed to rule out vascular causes.

## Conclusions

It is important to rule out sinister causes of RLN/vocal cord palsy with a focused clinical history, appropriate examination and endoscopy. If there is no obvious cause, a CT scan with contrast from the base of the skill to the costal margin of the thorax is indicated. Vascular causes of RLN/vocal cord palsy and KD as a differential for unilateral vocal cord palsy should be considered, with referral back to specialist cardiothoracic teams when symptoms change for patients with known vascular anomalies.
